# Genome-Wide Identification and Characterization of the *Salvia miltiorrhiza* Histone Deacetylase (HDAC) Family in Response to Multiple Abiotic Stresses

**DOI:** 10.3390/plants13050580

**Published:** 2024-02-21

**Authors:** Junyu Chen, Yuxin Ying, Lingtiao Yao, Zhangting Xu, Zhenming Yu, Guoyin Kai

**Affiliations:** 1 School of Pharmaceutical Sciences, Academy of Chinese Medical Sciences, Zhejiang Chinese Medical University, Hangzhou 310053, China; junyu_chen2023@163.com (J.C.); 19857992819@163.com (L.Y.); 15168104031@163.com (Z.X.); 2 College of Food and Health, Zhejiang A & F University, Hangzhou 311300, China; yingyuxin0123@163.com

**Keywords:** phytohormone, histone deacetylase (HDAC), expression profiles, genome-wide identification, phenolic acids, *Salvia miltiorrhiza*

## Abstract

*Salvia miltiorrhiza* is a plant commonly used in traditional Chinese medicine. Its material bases for treating diseases are tanshinones and phenolic acids, including salvianolic acids. Histone deacetylase proteins (HDACs) are a class of specific functional enzymes that interact with acetylation groups on the N-terminal lysine of histone proteins further regulate gene transcription through structural changes at the chromatin level. HDACs involved in the growth and development of various plants, and induced by plant hormones to regulate the internal environment of plants to resist stress, at the same time affect the accumulation of some secondary metabolites. However, the role of SmHDACs on the accumulation of salvianolic acid in *S. miltiorrhiza* remains unclear. In this study, 16 SmHDACs genes were identified from the high-quality *S. miltiorrhiza* genome, their physicochemical properties were predicted. In phylogenetic trees co-constructed with HDACs proteins from other plants, SmHDACs was divided into three subfamilies, each with similar motif and conserved domain composition. The distribution of the three subfamilies is similar to that of dicotyledonous plants. Chromosome localization analysis showed that SmHDACs genes were randomly located. Cis-acting element analysis predicted that SmHDACs gene expression may be related to and induced by various phytohormones, such as MeJA and ABA. By combining the expression pattern and co-expression network induced by phytohormones, we speculate that SmHDACs may further influence the synthesis of salvianolic acid, and identified SmHDA5, a potential functional gene, then speculate its downstream target based on the co-expression network. In summary, we analyzed the SmHDACs gene family of *S. miltiorrhiza* and screened out the potential functional gene *SmHDA5*. From the perspective of epigenetics, we proposed the molecular mechanism of plant hormone promoting salvianolic acid synthesis, which filled the gap in the subdivision of histone deacetylase in *S. miltiorrhiza* research, provided a theoretical basis for the culture and transformation of *S. miltiorrhiza* germplasm resources.

## 1. Introduction

*Salvia miltiorrhiza* is a Chinese traditional medicinal plant that has been used for thousands of years. It is rich in water-soluble component salvianolic acids and lipid-soluble component tanshinones [1,2], which is used to combat the increasing incidence of cardiovascular disease [3,4]. However, the wild resources of *S. miltiorrhiza* are decreasing continuously, which cannot meet the market demand. Therefore, it is urgent to innovate the cultivation technology of *S. miltiorrhiza*, especially to improve the content of medicinal components by metabolic engineering [5]. Salvianolic acid synthetic pathway has been improved with the efforts of researchers, simply put, the phenylpropanoid and tyrosine-derived pathways work simultaneously to generate two critical precursors, 4-coumaroyl-CoA and 3, 4-dihydroxyphenyllactic acid, consequently under the catalysis of rosmarinic acid synthetase (RAS) and cytochrome P450-dependent monooxygenase (CYP98A14) to product rosmarinic acid, but the subsequent synthesis of Salvianolic acid B is uncertain [6,7,8].

Based on the above synthetic pathways, the regulation mechanism of salvianolic acid synthesis has been studied extensively. Various signaling pathways specifically activate certain transcription factors for activating target genes to promote salvianolic acid synthesis, and this kind of metabolic mechanism has been extensively studied. For example, JA signaling pathway induced by jasmonic acid (JA) has been proved to significantly increase the content of salvianolic acid [9]. In-depth study on JA pathway found that some transcription factors activated by JA pathway can enhance the transcription of salvianolic acid key enzyme genes, such as basic helix-loop-helix (bHLH), R2R3-MYB transcription factor MYB76, MYB1, MYB2 [2,10,11]; APETALA2/ERF (AP2/ERF) transcription factor ERF115 [12]. However, in addition to transcription factors, there are many other factors affecting gene expression such as post-translational modifications (PTMs). These modifications occur after transcription and work through structural changes at the chromatin level, known as epigenetics.

DNA wraps around octamer protein core of histones (two copies of H2A, H2B, H3, H4) to form a nucleosome [13]. N-terminal tails of histone proteins protrude from nucleosome, where PTMs events occurred, such as acetylation, methylation and phosphorylation [14]. Among them, acetylation of histone lysine affects the structure of the nucleosome, thus affect the expression of the gene [15]. The level of histone acetylation is a dynamic equilibrium process, which is dominated by histone acetyltransferase and histone deacetylases (HDACs) [16], when the balance upset, histone acetylation levels rise/fall, generally means that gene transcription is activated/suppressed [17]. In plants, HDACs are divided into three subfamilies, Reduced Potassium Dependency 3/histone deacetylase 1 (RPD3/HDA1), Silent Information Regulator 2 (SIR2) and the plant-specific histone deacetylase 2 (HD2) [18]. The effects of histone acetylation on plants are comprehensive, including the regulation of plant growth and development, as well as various secondary metabolism. For example, in *Arabidopsis thaliana*, PIF1/PIF3 interact with HDA19 and MED25 to mediate transcriptional repression in the phytochrome signaling pathway [19]; HDA5 is involved in the control of cytosolic metabolism and cell function [20]. Moreover, HDACs showed sensitivity to various phytohormones in recent reports, OsHDA710 in rice is sensitive to ABA and JA, which regulates the expression of salt-tolerant genes [21]; AtHDA6 in *Arabidopsis thaliana* direct deacetylation of TOPLESS protein (JA pathway suppressor), under JA induction, AtHDA6 expression increases and breaks homeostasis, the inhibitory effect of TOPLESS is weakened thus activating JA pathway [22]. It is known that increased expression of DNA methyltransferase related genes contributes to increased DNA methylation levels during root growth of *S. miltiorrhiza* and changes the accumulation of secondary metabolites [23]. However, the role of histone deacetylation and expression of HDACs in salvianolic acid accumulation in *S. miltiorrhiza* are still unclear.

In this study, we identified SmHDACs (*Salvia miltiorrhiza* Histone Deacetylase) genes from high-quality genomes for bioinformatics analysis. Muti-transcriptome data were used to analyze the expression profiles of SmHDACs under the influence of hormones, further potential functional genes were screened. Our results confirmed that the expression of SmHDACs gene changes under various stresses, indicating that histone deacetylation plays an important role in *S. miltiorrhiza* under stress. The potential role of SmHDACs in the synthesis of salvianolic acid were further discussed. These conclusions are of great significance for further research on the epigenetics of *S. miltiorrhiza*.

## 2. Results

### 2.1. Identification of SmHDACs

Through HMMER server, there are 12 RPD3/HDA1 subfamily and 2 SIR2 subfamily SmHDACs, respectively. By BLAST of AtHDTs, two SmHDTs are found, both of which contain NPL conserved domains verified by HMMER server. All the above 16 SmHDACs passed the conserved domain integrity verification, and named SmHDA1-12, SmSRT1-2, and SmHDT1-2 according to subfamily affiliation, respectively, as shown in Table 1. Their protein size ranges from 284 to 776 (aa), molecular weight from 30.699 to 85.280 (kDa), isoelectric point from 5.01 to 9.40. Other physical and chemical properties are shown in Table 1. Subcellular localization prediction found that most SmHDAs were predicted in the nucleus and cytoplasm, while all SmSRTs were predicted in chloroplasts, may be able to act on proteins that respond to light. Only SmHDT2 is predicted to be located in the vacuole (Table 1).

### 2.2. Phylogenetic Tree of SmHDACs

**Figure 1:**
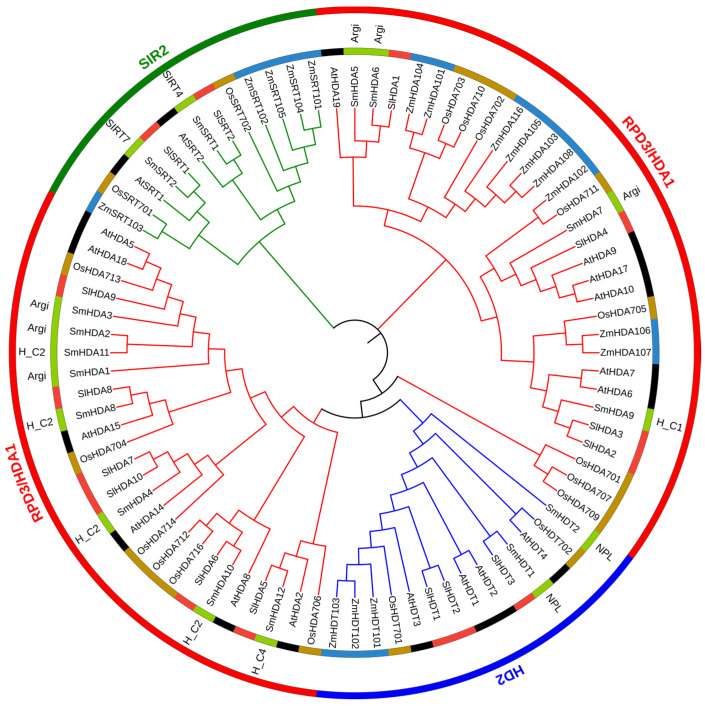
Phylogenetic tree of the SmHDACs in *Salvia miltiorrhiza* and in four other plant species. The color of the inner ring represents the species: green, *S. miltiorrhiza*; red, *S. lycopersicum*; black, *A. thaliana*; brown, *O. sativa*; blue, *Z. mays*. Annotate the conserved domain in SmHDACs above *S. miltiorrhiza*. The outer ring represents the subfamily: red, RPD3/HDA1 subfamily; Blue, HD2 subfamily; Green, SIR2 subgroup. Between the two rings, the comments on *Salvia miltiorrhiza* are conserved domain types, which are: Agri, Arginase_HDAC, cl17011; H_C1, HDAC_classII_1, cd09996; H_C2, HDAC_classII, cd09992; H_C4, HDAC_classIV, cd09993, so far, they are all conserved domains owned by the RPD3/HDA1 subfamily; NPL, cl03870, a conservative domain unique to HD2 subfamily; SIRT4, cd01409, SIRT7, cd01410, a conservative domain unique to the SIR subfamily.

SmHDACs and HDACs from four species (*Arabidopsis thaliana*, *Oryza sativa*, *Zea mays*, *Solanum lycopersicum*), were used to build phylogenetic tree, the conservative domain information of SmHDACs is also added, as shown in Figure 1. The grouping of all proteins was exactly as expected, and the three subfamilies were completely separated. The largest subfamily of SmHDACs is RPD3/HDA1 subfamily, with a total of 12. The number of SIR2 and HD2 subfamilies is equal, both with 2. The conserved domains of each subfamily also conform to the initial description, that is, the SIRT4 and SIRT7 domain is owned specifically by the SIR2 subfamily, the NPL domain is owned specifically by the HD2 subfamily, and the HDA subfamily contains multiple HDAC domains, such as Arginase_HDAC superfamily (Accession = cl17011), HDAC_classII (Accession = cd09992) et al., and the aggregation of SmHDACs is independent of the conserved domain. Different from other species, there are more HDACs in the HD2 and SIR2 subfamilies in *Z. maize*, which shows that monocotyledonous plants and dicotyledonous plants have different evolutionary trends of HDACs.

### 2.3. Gene Structure and Motif Analysis of SmHDACs

**Figure 2:**
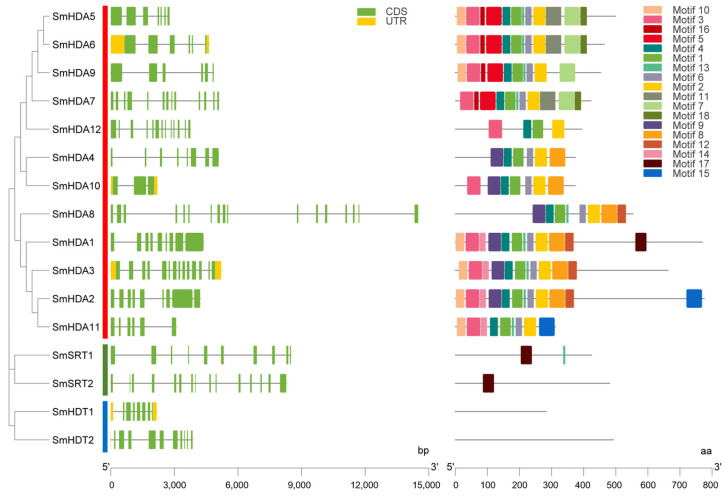
Analysis of gene structure and motifs of the SmHDACs. All motifs of SmHDACs and their gene locations were labeled and classified according to subfamilies. Motifs of the same subfamily have similarities. SmHDTs does not contain motif. Motif logo as show in Figure S1.

The gene structure and motif information of SmHDACs are shown in Figure 2. There were no significant association between gene structure and SmHDACs grouping. A total of 17 motifs have been excavated, named Motif 1-17. Their distribution is obviously related to grouping. SmHDACs include all motif 1-17, clustered SmHDACs have similar motifs (Specific motif sequence information is shown in Figure S1). Interestingly, the motif analysis results of SmHDA5 and SmHDA6 are consistent, they are also grouped into clusters in phylogenetic tree, indicating that their conserved domains are similar, which indicated they may further lead to functional similarities. A similar situation also occurs for SmHDA1-3, and the results showed that they differ only in the terminal motifs. On the other hand, SmHDTs does not contain any motifs, a similar pattern has been observed in *Dendrobium officinale* SRTs [24].

### 2.4. Chromosome Localization and Collinearity Analysis of SmHDACs

**Figure 3:**
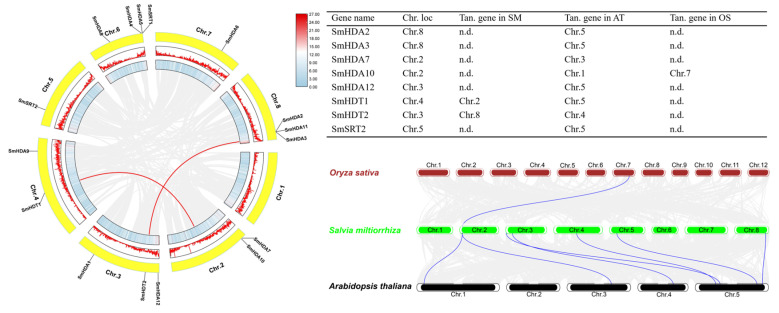
Chromosome localization and collinearity analysis of *SmHDACs*. The 16 genes were randomly distributed on 8 chromosomes, and tended to be in the locations with high gene density. *SmHDT1* and *SmHDT2* have collinear genes and are labeled with red lines. The collinear genes between multiple species are shown as blue lines, and all the results are counted. Ten. Gene in AT means collinear genes in *A. thaliana*, SM means *S. miltiorrhiza*, OS means *O. sativa*. n.d. indicates that there are no collinear genes in the species.

The distribution of 16 SmHDACs on the chromosome map is shown in Figure 3. Meanwhile, we also labeled the density of chromosomes and the collinearity analysis among SmHDACs. In order of number, chromosome (Chr.) 6, 8, 3, 4, 2, 5, 7 distributed 4, 3, 3, 2, 2, 1, 1 gene, respectively, while SmHDACs were not distributed on Chr.1. *SmHDA4*, *5*, and *SmSRT1* are clustered on Chr.6, and *SmHDA2*, *3*, *11* are clustered on Chr.8. Most SmHDACs are found in areas with high gene density, and histone deacetylated proteins may play a role in many gene clusters. *SmHDT1* and *SmHDA2* have collinear genes on Chr.2 and Chr.3, respectively, suggests that these genes may have duplicated along the way to take on important functions. Collinearity analysis with other species showed that SmHDACs had collinear gene with *A. thaliana*, but there were few genes in rice genome, indicating that *S. miltiorrhiza* and *A. thaliana* were closely related.

### 2.5. Cis-Acting Elements Analysis of SmHDACs

**Figure 4:**
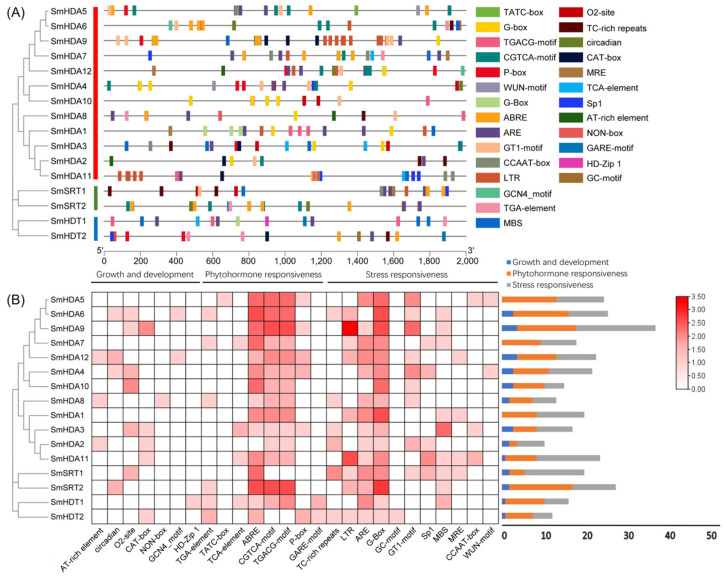
Cis-acting element analysis of SmHDACs. (**A**), The position of each CAEs on the SmHDACs promoter. (**B**), The statistical heatmap of each CAEs on the promoter of SmHDACs, all CAEs were divided into three parts: growth and development, phytohormone responsiveness and stress responsiveness, among which the number of CAEs responding to phytohormone MeJA was the largest.

The upstream 2000 bp of a gene is regarded as a promoter region, it was extracted from the genome for cis-acting element (CAEs) analysis. We visualized the distribution of CAEs of 16 SmHDACs on promoters and categorized them as growth and development, phytohormone responsiveness, and stress responsiveness, as shown in Figure 4. A total of 27 CAEs were randomly distributed on the promoters of all SmHDACs, involving many events such as plant growth and development, phytohormone responsiveness and so on (Figure 4A). Further study found that the number of CAEs related to phytohormone response was the highest, while the number related to growth and development was the lowest (Figure 4B). G-Box, CGTCA-motifs and TGACG-motifs related to MeJA response accounted for 37.3% of all CAEs, indicating that SmHDACs may be strongly associated with phytohormone MeJA. On the other hand, a total of 46 ABRE were found, suggesting that SmHDACs may play a role in responding to ABA. In addition, SmHDACs may respond to gibberellin (P-box, TATC-box, GARE-motif), salicylic acid (TCA-element), and auxin (TGA-element). SmHDACs may also play a role in the following stresses: low temperature (LTR), hypoxia (ARE), light (GT1-motif, G-box, sp1, MRE), drought (MBS), and mechanical injury (WUN-motif), Low temperature and hypoxia are the most likely stressors to induce *SmHDACs* expression. Under these two conditions, SmHDACs protein may enhance its histone deacetylation function. SmHDA5, 6, 9 and SmSRT2 have a large number of CAES, which may be active in plant metabolic regulation species, and may be potential functional genes.

### 2.6. Tissue-Specific Analysis of SmHDACs

**Figure 5:**
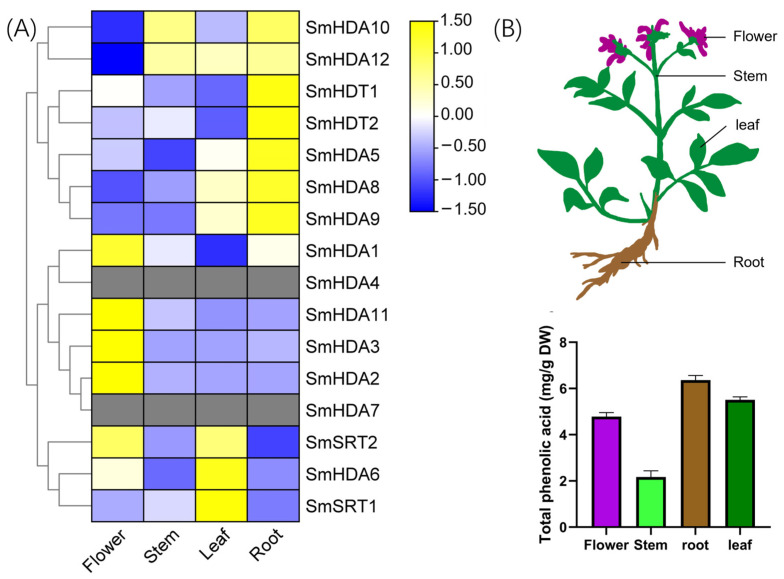
Tissue-specific analysis of SmHDACs. (**A**), Total phenolic acid content in roots, stems, leaves, and flowers; (**B**), Schematic diagram of *S. miltiorrhiza* tissues and expression profile of SmHDACs genes in flowers, leaves, stems and roots. DW, dry weight.

The expression of genes in individual plant tissues is significantly correlated with their functions. Here, we analyzed the tissue specificity of *SmHDACs* in roots, stems, leaves, and flowers, as shown in Figure 5A. Half of the genes are highly expressed in the root, which may be involved in the synthesis of salvianolic acid. *SmHDA6* and *SmSRT1*, *2* are highly expressed in leaves, which may be related to photomorphogenesis or anthocyanin synthesis. *SmHDA1*, *2*, *3*, *11* and *SmSRT2* are highly expressed in flowers, which may play a role in plant maturation. Some of the functions of these hypotheses can be matched with the results of CAEs analysis (Figure 4A). For example, *SmHDA6* and *SmSRT2* are highly expressed in leaves and contain GT1-motif (light responsiveness), indicating that they may participate in plant photosynthesis. Total phenolic acid content was highest in roots (Figure 5B), suggesting that SmHDACs such as *SmHDA10*, *12*, *5*, *8*, *9* and *SmHDT1*, *2*, which are specifically expressed in roots, may be involved in the synthesis of phenolic acids.

### 2.7. Induction of SmHDACs Expression Patterns by Each Inducer

**Figure 6:**
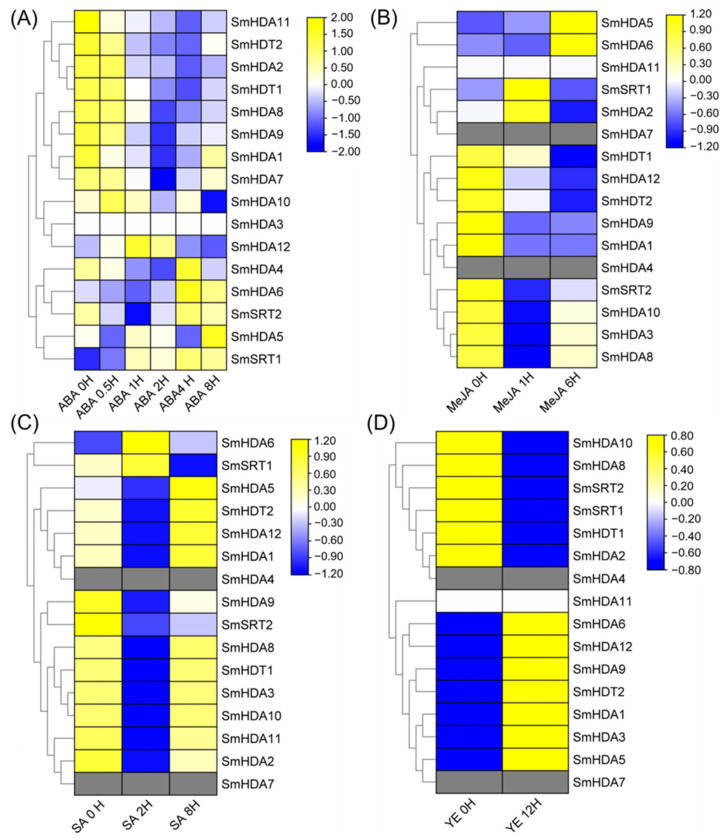
Analysis of the expression profile of the SmHDACs induced by exogenously applied phytohormones. (**A**) Expression of SmHDACs induced by ABA at six time points (0, 0.5, 1, 2, 4, and 8 h); (**B**) Expression of SmHDACs induced by MeJA at three time points (0, 6, and 12 h); (**C**) Expression of SmHDACs induced by SA at three time points (0, 2, and 8 h); (**D**) Expression of SmHDACs induced by YE at two time points (1 and 12 h). ABA, abscisic acid; MeJA, methyl jasmonate; SA, salicylic acid; YE, yeast extract. Different elicitation was assessed at different time point.

Since CAEs analysis indicated that the promoters of SmHDACs may have CAEs in response to various phytohormones, we analyzed the expression pattern of SmHDACs under multi-medium inducer treatment by accessing the transcriptome database, include ABA, MeJA, SA, YE (Figure 6).

Under Abscisic acid induction, *SmHDA3* remained unexpressed, while *SmHDA5*, *6*, and *SmSRT1*, *2* tended to be activated by ABA (Figure 6A). The expression of SmHDACs decreased in more than half of the patients, indicating the overall inhibition of ABA on SmHDACs.

Methyl jasmonate (MeJA) is one of the metabolites that plants biosynthesis when Subject to biological or abiotic stress, it is an important hormone in *S. miltiorrhiza* which has been shown to promote the accumulation of salvianolic acid [9]. According to the results of MeJA induction, the expressions of *SmHDA2*, *5*, *6* and *SmSRT1* were significantly up-regulated, while others were down-regulated, and *SmHDA11* was not expressed (Figure 6B). Among them, only *SmHDA5* and 6 were continuously activated by MeJA, indicating that they may play a role in the JA response pathway related proteins under MeJA induction.

Salicylic acid can inhibit almost all SmHDACs except *SmHDA6* and *SmSRT1* (Figure 6C). After treated with yeast extract that was shown to induce the production of specialized metabolites [9], half of the genes, including *SmHDA6*, were activated and the other half were suppressed (Figure 6D). According to the above conclusions, it can be seen that the most likely functional gene of *SmHDA5*, *6* in SmHDACs is the core histone deacetylation protein of *S. miltiorrhiza* in response to stress.

### 2.8. Co-Expression Network Analysis by MeJA and ABA Treatment

**Figure 7:**
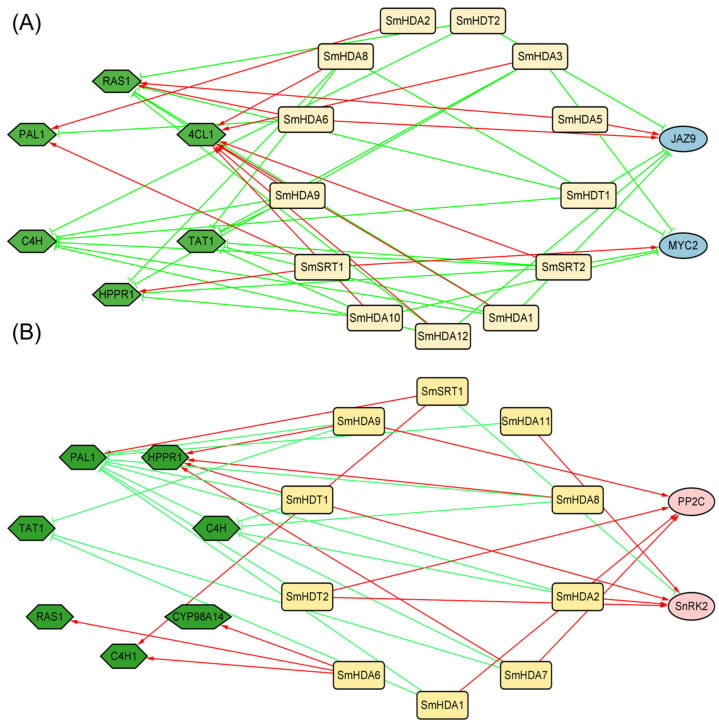
Co-expression network analysis of the *SmHDACs*. After phytohormony treatment, a co-expression network between all 16 *SmHDACs* and the critical salvianolic acid biosynthetic genes were carried out using Cytoscape software v3.9.1 (|r| > 0.8, *p* < 0.05). (**A**) *SmMYC2* and *SmJAZ9* of MeJA pathway. (**B**) *SnRK2* and *PP2C* of ABA pathway. Four core transcription factors or core protein coding genes on two phytohormone pathways were added for further downstream target prediction. Red/Green arrows indicate positive/negative correlations.

In order to further explore the molecular mechanism of how SmHDACs mediates hormone signaling pathway to regulate salvianolic acid synthesis, we established a co-expression network of salvianolic acid pathway synthase genes and *SmHDACs* by MeJA and ABA treatment (Figure 7). At the same time, the core transcription factors of JA pathway and ABA pathway were also added, as a preliminary prediction of downstream targets of SmHDACs (Figure 7A). At the three time points after MeJA treatment, the highest number of SmHDACs were close to and positively correlated with the expression trend of the key enzyme gene *Sm4CL1*, suggesting that *Sm4CL* may be the final target for *SmHDAC* to affect salvianolic acid synthesis. *SmJAZ9* is negatively correlated with more *SmHDACs*, may be one of the main targets of histone deacetylation.

Under ABA induction, *SmPAL1* was inversely associated with the most *SmHDACs* and most likely the ultimate target (Figure 7B). Compared with PP2C, SnRK2 is more likely to be the downstream target of *SmHDACs*, which may be deacetylated by *SmSRT1*, resulting in repression of gene expression. ABA hormones are often biosynthesized by plants in response to stresses such as drought, low temperature [25,26], among which *SnRK2* acts as an intermediate transcription factor during the occurrence of these stresses. *SmHDA2*, *11* and *SmHDT1*, *2* were positively correlated with the expression of *SnRK2*, indicating that they may play a role in the stress of *S. miltiorrhiza*, while *SmSRT1* is negatively correlated. It may be the upstream acting protein of *SnRK2*.

## 3. Discussion

Histone deacetylation is an important post-translational modification that regulates gene expression by regulating the acetylation level of histone terminal lysine. After the deacetylation of histones terminal lysine by HDACs, the structure tends to compress, making it difficult for DNA to be transcribed, thus inhibiting and even silencing the genes [14]. It has been widely studied in plants and can be divided into three subfamilies: RPD3/HDA1, SIR2 and HD2 [18]. In this study, 16 SmHDACs proteins were discovered from the high-quality genome of *S. miltiorrhiza* [27], and named according to subfamilies, with 12 RPD3/HDA1,2 SIR2 and 2 HD2, respectively (Table 1), which ratio was similar to other dicotyledonous plants such as *A. thaliana*, *O. sativa*, et al., the RPD3/HDA1 subfamily of HDACs is dominant in numbers, and this pattern is also reflected in more species [28,29]. However, although the RPD3/HDA1 subfamily is the most numerous, many of the genes in this subfamily function were similar [30]. According to motif analysis (Figure 2), motifs of SmHDAs were more conserved, while motifs were not detected by SmSRTs, indicating that the number of SmSRT mutations during evolution may be more. From the perspective of collinear analysis (Figure 3), *SmHDT1* and *SmHDA2* have collinear genes on Chr.2 and Chr.3, respectively, suggests that SmHDTs may be copied by other genes during evolution, and may play an important role in the adaptation of *S. miltiorrhiza* to certain environmental changes. Although HDACs functions in so many different ways, unfortunately, the molecular mechanisms of phytohormones promote salvianolic acid accumulation on histone deacetylation have not received sufficient attention.

Under biological or abiotic stress, *S. miltiorrhiza* may coordinate homeostasis by secreting phytohormones and other ways, thereby some important secondary metabolites are synthesized in large quantities during this process, including salvianolic acid, an important medicinal ingredient [1,10]. In this study, the content of total phenolic acid in various tissues of *S. miltiorrhiza* was quantitatively determined, results demonstrated that the root is the main accumulation site of salvianolic acids (Figure 5B), therefore, the relationship between SmHDACs with higher expression in the root and salvianolic acid synthesis is most valuable to discuss. Compared with other tissues, *SmHDT1*, *2* and *SmHDA5*, *8*, *9*, *10*, *12* have the highest expression levels in roots, and the proteins encoded by them may be the most active in roots. The amount of salvianolic acid was increased by ABA, and the expression of SmHDACs activated at certain time nodes under ABA treatment included *SmHDA1*, *4*, *5*, *6*, *7*, *10*, *12* and *SmSRT1*, *2*. Combining the two expression profiles, we can preliminatively conclude that SmHDA5, 10, and 12 are potential ABA-induced genes that increase salvianolic acid content. On the other hand, co-expression networks can also help search for potential functional genes (Figure 6). For example, *SmSRT2* is the only gene negatively related to *SnRK2*, which is a positive regulator of ABA pathway and plays a central role in the entire transcriptional network. Moreover, *SmSRT2* was induced by ABA, suggested that *SmSRT2* may be induced after ABA secretion, one of the restrictors of this pathway, and may directly deacetylate *SnRK2*. Similarly, the expression of *SmHDA2*, *5*, *6* and *SmSRT1* was upregulated under MeJA induction, among which *SmHDA5* was the root gene with high expression, therefore it can be preliminarily speculated that *SmHDA5* might have an effect on salvianolic acid synthesis. It is worth noting that *SmHDA5* also positively correlated with the expression of *SmJAZ9*. In previous reports, *SmJAZ9* was shown that it can form the complex with *SmMYB76*, further inhibit the expression of the synthetic gene *SmRAS1*, SmMYB76 was also associated with *SmHDA5* [2]. The addition of SmHDA5 may provide a new idea for the molecular mechanism of SmMYB76 inhibiting salvianolic acid synthesis. According to the above reasoning, SmHDA5 may have multiple downstream targets, which can inhibit the expression of SmMYB76, SmJRB1 [1] and SmJAZ9 through histone deacetylation, weaken the inhibitory effect of the latter on synthase, and promote the synthesis of salvianolic acid.

Phytohormone is a special metabolite secreted by plants to fight against external biological or abiotic stress, which can maintain the homeostasis of the plant internal environment [31]. In the CAEs analysis (Figure 4), we found that the response to plant hormones may be the main functional pathway of SmHDACs, compared with plant growth and development and stress responsiveness. CAEs related to phytohormone responsiveness dominated in number, especially in response to MeJA and ABA. It is worth noting that both MeJA and ABA have been reported that can promote medical compounds salvianolic acid accumulation [9,32], the complex molecular mechanisms involved have been extensively studied. In addition to the role of transcription factors, SmHDACs provide a potential molecular mechanism at the epigenetic level. SmHDACs may have deacetylated effect on some key transcription factors in plant hormone signaling pathway, thus regulating the whole pathway, such mechanisms have been resolved in other plants. For example, in tea, CsHDA6 deacetylates histone proteins of CsMYC2, affecting the latter’s ability to enhance the transcription of CsAFS and inhibiting the accumulation of α-farnesene in tea, while the intervention of MeJA inhibits this effect [33], a similar mechanism occurs in CsHDA2 and ultimately affects the pest resistance of tea [34]. Furthermore, OsHDA710 has been shown to enhance the sensitivity of rice to ABA, which is reflected in the salt tolerance of rice [21]. Even further studies have found that HDACs can directly act on some proteins, but not histones, for deacetylation, such as Arabidopsis HDA6 can deacetylate TPL protein, which is the restriction of JA pathway activation, HDA6 and GCN5 jointly maintain the acetylation homeostasis of TPL protein, thereby affecting the expression of subsequent transcription factors on the JA pathway, such as MYC2, JAZ, et al. [22]. To explore the role of SmHDACs in response to phytohormones, and to look for potential functional genes, we used a series of bioinformatics analysis methods such as CAEs analysis, phylogenetic tree, and induced expression pattern. Genes assigned to one branch of the phylogenetic tree may have similar functions, for example, AtHDA19 is a protein with multiple functions, which can regulate photomorphogenesis and seed dormancy et al. [35,36], and can be regulated by MeJA and ABA [37,38], the homologous *SmHDA5*, *6* may also play the similar role. CAEs analysis showed that *SmHDA5*, *6* were one of the SmHDACs with the largest number of hormone-responsive elements (Figure 4), and the expression of these two genes were activated under the induction of MeJA and ABA (Figure 6), indicating that they are likely to be able to respond to these two phytohormones to play more subsequent functions, this confirms previous predictions of their function by the phylogenetic tree. Similarly, it is speculated that the AtHDA6 homologous SmHDA9 may have the function of non-histone deacetylation [22], however, it is inhibited by MeJA (Figure 6), suggesting that the target of SmHDA9 may be a negative regulator in the pathway. SmSRTs is also a noteworthy gene. In *A. thaliana*, AtSRTs is usually associated with the growth and development process. For example, AtSRT2 can regulate the salt-stress resistance of Arabidopsis seed during germination [30], AtSRT1 can regulates primary metabolism by modulation of AtMBP-1 transcriptional activity [39]. Their homologous SmSRT1, 2 probably similar to AtSRTs. The expression levels of them changed little under the treatment of TSA, and the changes induced by hormones were not obvious, indicating that their expression was stable. In general, the SmHDACs family may play a role in various growth stages of *S. miltiorrhiza*, and regulate the expression and affect the function of some transcription factors under the induction of plant hormones.

## 4. Materials and Methods

### 4.1. Plant Materials, Hormonal/Elicitor Treatment

Three-mouth-old aseptic plant of *S. miltiorrhiza* cultured in a greenhouse at 26 °C with Murashige and Skoog medium, exposure to 16-h light/8-h dark. The hairy roots of *S. miltiorrhiza* were obtained by infecting leaves or stems from aseptic plant with *Agrobacterium rhizogenes* strain C58C1 (pRi A4) (Weidi Biotechnology Co., Shanghai, China), then subcultured in half-strength MS medium. The young hairy roots were cut and cultured in liquid medium with persistent darkness at 25 °C and 120 rpm for 50 days, therewith treated with various exogenous inducers. Additional transcriptome data for this article can be downloaded by BioProject access numbers: PRJNA703309 (ABA), PRJNA393563 (MeJA), PRJNA301529 (SA), and PRJNA393563 (YE) [40].

Mature one-year raw *S. miltiorrhiza* planted in the greenhouse of our laboratory was used to determine the total phenolic acid content of each tissue. The roots, stems, leaves and flowers of *S. miltiorrhiza* were collected, dried in an oven at 65 °C, and ground into powder for subsequent experiments.

### 4.2. Identification of the HDACs Family in S. miltiorrhiza

The newly assembled annotated high-quality genomic data is used to mine SmHDACs [27]. To get the subfamily of RPD3/HDA1 and SIR2, Hist_deacetyl domain (PF00850) and SIR2 domain (PF02146) were utilized as query to search against *S. miltiorrhiza* protein database by Hidden Markov Model (HMM) function module of TBtools software (v2.026) [41], e-value > 0.05 is used as a threshold filter. Moreover, HD2 subfamily was discovered by BLSAT to AtHDT (AT3G44750, AT5G22650, AT5G03740, AT2G27840) protein sequence in model plant Arabidopsis thaliana, the result is compared to verify the NPL domain (PF17800) by HMM. All the SmHDACs obtained above were verified by Conserved Domain Database (http://www.ncbi.nlm.nih.gov/Structure/cdd/wrpsb.cgi, (accessed on 5 August 2023)) to ensure their integrity, and the incomplete sequences in the conserved domain were deleted. Finally, 16 SmHDACs were identified (Table S1). Furthermore, the physical and chemical properties of SmHDACs were predicted by ExPASy tool (https://web.expasy.org/, (accessed on 6 August 2023)), and the subcellular localization information was predicted by Plant-mPLoc predictor (http://www.csbio.sjtu.edu.cn/bioinf/plant-multi/, (accessed on 6 August 2023)).

### 4.3. Phylogenetic Tree, Motif and Gene Structure Analysis of SmHDACs

To understand the evolutionary relationship of SmHDACs, HDACs of *Arabidopsis thaliana* collected from TAIR database (https://www.arabidopsis.org/, (accessed on 2 August 2023)), *Oryza sativa*, *Zea mays*, and *Solanum lycopersicum* collected from Phytozome database (https://phytozome-next.jgi.doe.gov/, (accessed on 20 July 2023)) (the protein sequence information is shown in Table S2) were aligned by ClustalW and construct phylogenetic tree by MEGA 11.0 software (https://megasoftware.net/, (accessed on 15 June 2022)) with Maximum likelihood algorithm. iTOL online tool (https://itol.embl.de) was employed for modifying the phylogenetic tree. The analysis of conserved motif was implemented by MEME tool (https://meme-suite.org/, (accessed on 10 August 2023)), with parameters as motif width 6–50 and max number is 20. The visualization of conserved motifs and gene structure of SmHDACs were employed by TBtools [41].

### 4.4. Chromosome Localization and Collinearity Analysis of SmHDACs

The distribution of SmHDACs at the chromosomes was analyzed through the annotation information. To explore gene duplication information between SmHDACs, collinearity analysis of members was performed by MCScanX [42] and visualized by Tbtools [41].

### 4.5. Cis-Acting Elements Analysis of SmHDACs

In order to search for the possible molecular mechanism of SmHDACs, the cis-acting elements on their promoters were analyzed. The upstream 2000 bp long sequences of SmHDACs proteins were extracted from S miltiorrhiza genome, then input into PlantCARE (http://bioinformatics.psb.ugent.be/webtools/plantcare/html/, (accessed on 13 August 2023)) [43] to predict potential cis-acting elements.

### 4.6. Organ-Specific and Each Inducer Treated Expression Pattern of SmHDACs

In order to study the ability of SmHDACs to respond to stress, we obtained multiple transcriptome data and analyzed their expression profiles. All SmHDACs were identified in muti-transcriptome by BLAST. The transcriptome of four organs (leaf, flower, root, stem) of *S. miltiorrhiza* were used for organ-specific analysis. The results are visualized by heatmap. In order to search for potential downstream targets of SmHDACs, key enzyme gene in the salvianolic acid synthesis pathway and core transcription factor genes (Table S3) in the MeJA pathways and ABA pathway were used as co-expression networks with SmHDACs, correlation coefficients test (Pearson’s coefficients, take its absolute value) >0.8 is considered as associated, the result is presented via cytoscape 3.9.1 software.

### 4.7. Determination of Total Phenolic Acid

To investigate the optimum tissue for the accumulation of total phenolic acid, roots, stems, leaves, and flowers of one-year-old *S. miltiorrhiza* were sampled. Afterwards, 100 mg of dry powder were obtained through mSini-pulverizer (Xinda Machinery Co., Jiangyin, China), extracted with an ultrasound-assisted method, and utilized 10 mL 80% ethanol solution as a solvent. Total phenolic acid content of four different tissues were quantitatively measured by an improved Folin-Ciocalteu method. In brief, 1 mL of alcohol extract solution was evenly mixed with 5 mL of 0.1 M Folin-Ciocalteu reagent (Solarbio Science & Technology Co., Beijing, China), 4 mL of 20% (*m*/*v*) Na_2_CO_3_ solution within 60 s, and keep the mixed solution away from light for 1 h. Gallic acid (Sigma-Aldrich, St. Louis, MO, USA) was utilized as reference for a standard curve (y = 8.068 x + 0.015, R^2^ = 0.999). The content of total phenolic acid was photometrically detected at 760 nm with UV spectrophotometer (Metash Instruments Co., Shanghai, China), and expressed as mg of gallic acid equivalents per 100 mg of dry weigh (DW).

## 5. Conclusions

In this paper, 16 SmHDACs were obtained from the high-quality genome of *Salvia miltiorrhiza*, and their biological characteristics were analyzed, including phylogenetic tree, cis-acting element analysis, prediction of subcellular localization, and collinearity analysis. The above analysis suggests that SmHDACs respond to a variety of phytohormones, and further affect the accumulation of salvianolic acid, the medicinal component of *S. miltiorrhiza*. The expression profiles of *SmHDACs* under various inducers were used to evaluate the ability of *SmHDACs* to respond to stress. Combined with the co-expression analysis network and previous reports, several potential functions of SmHDACs were speculated, and a molecular mechanism of SmHDA5 mediated JA pathway in promoting salvianolic acid accumulation was further proposed. This study provides a strategy to improve the quality of *S. miltiorrhiza* from the perspective of epigenetic histone deacetylase.

## Figures and Tables

**Table 1:** Physiochemical properties of the *SmHDACs*.

Name	Size/aa	Molecular Weight/kDa	PI	GRAVY	Aliphatic Index	Instability Index (II)	Subcellular Localization
SmHDA1	770	83.873	5.81	−0.249	83.14	40.40	Nucleus
SmHDA2	776	85.280	6.39	−0.385	84.36	53.08	Nucleus
SmHDA3	663	73.814	5.27	−0.248	85.78	49.16	Chloroplast and Nucleus
SmHDA4	374	40.619	5.69	−0.097	87.19	37.65	Nucleus
SmHDA5	499	56.276	5.27	−0.489	75.99	38.73	Cytoplasm and Nucleus
SmHDA6	464	52.436	5.15	−0.544	72.87	38.75	Cytoplasm and Nucleus
SmHDA7	423	47.742	5.01	−0.365	81.82	31.80	Cytoplasm and Nucleus
SmHDA8	554	60.571	5.68	−0.229	79.82	41.16	Nucleus
SmHDA9	453	51.454	5.82	−0.513	76.16	47.61	Nucleus
SmHDA10	374	40.411	5.38	−0.124	86.55	34.18	Nucleus
SmHDA11	313	35.305	6.74	−0.416	86.36	42.16	Nucleus
SmHDA12	394	43.173	7.21	−0.112	94.85	36.19	Nucleus
SmSRT1	425	47.983	9.40	−0.450	77.13	45.82	Chloroplast
SmSRT2	481	53.433	9.34	−0.180	89.58	39.70	Chloroplast
SmHDT1	284	30.699	5.07	−1.059	50.28	46.80	Nucleus
SmHDT2	493	53.822	5.09	−0.992	61.08	47.81	Vacuole and Nucleus

## Data Availability

The datasets analyzed during the current study are available at the NCBI under the accession number PRJNA703309, PRJNA393563, PRJNA301529, and PRJNA393563 for the ABA, MeJA, SA and YE treatments [40], respectively. All methods were carried out in accordance with relevant guidelines and regulations. All experimental studies on plants were complied with relevant institutional, national, and international guidelines and legislation.

## Supplementary Materials

The following supporting information can be downloaded at: https://www.mdpi.com/article/10.3390/plants13050580/s1, Figure S1: Seventeen motif logos in SmHDAC protein. Table S1: Protein sequence of all SmHDACs; Table S2: Protein sequences used to construct the phylogenetic tree; Table S3: Gene ID used to construct a co-expression network.
