# Supplementary material for: Genome-Wide Identification and Characterization of the Salvia miltiorrhiza Histone Deacetylase (HDAC) Family in Response to Multiple Abiotic Stresses
Source: Plants (Basel). 2024 Feb 21;13(5):580. doi: 10.3390/plants13050580 (PMC10935284; doi:10.3390/plants13050580)
Supplement: Supplementary file 1 [file plants-13-00580-s001.zip › plants-2838663-supplementary.pdf]

Genome-Wide Identification and Characterization of the *Salvia miltiorrhiza* Histone Deacetylase (HDAC) Family in Response to Multiple Abiotic Stresses

Junyu Chen [1,†], Yuxin Ying [2,†], Lingtiao Yao [1], Zhangting Xu [1], Zhenming Yu [1,*] and Guoyin Kai [1,*]

[1] School of Pharmaceutical Sciences, Academy of Chinese Medical Sciences, Zhejiang Chinese Medical University, Hangzhou 310053, China

[2] College of Food and Health, Zhejiang A & F University, Hangzhou 311300, China

[*] Correspondence: yuzhenming@zcmu.edu.cn (Z.Y.) ; kaiguoyin@163.com (G.K.)

† These authors contributed equally to this work.

Table S1 Protein sequence of all SmHDACs.

Table S2 Protein sequences used to construct the phylogenetic tree.

Table S3 Gene ID used to construct a co-expression network.

Figure S1 Seventeen motif logos in SmHDAC protein.

Table S1 Protein sequence of all SmHDACs.

Gene name	Gene ID	Protein sequence
SmHDA1	SMILT033186.1	MSRNNDSVSGRDGRVGLIFDERMCKHYAPAEEDHPECPNRIRVIWNLLDSSGLAKRCIILKAKDAEYNHLALVHTKKHISLIKNISSRNTQSKRERIAVKFDSIYFNEGSTEAACLAAGSVIEAAEKVASGEIDSAFAIVRPPGHHAKEGEPMGFCLYNNVAIAASYLLNERQELGINKILIVDWDVHHGNGTQNMFYKDPRVLVFSVHRHDFGTFYPSGNGGSYIMTGEGEGAGHNINVPWEHGQCGDADYLAVWDHILIPVAKEFKPDIIISAGFDAAKGDPLGGCCVSPNGYSVMLHKLMEFAGGKIVMALEGGYNLNSTASSALACVQALLHNEPPNVVSQARPFASTWRVIQQVCGVLRPYWPVLARIDNNHPTESDPVTSPHVPEKGLLRPCNMSSIKKSTGQSSGEADSLQTLAGDIGSGAGADKPANFPRPLRNLTGAECQAQMEAQIASSGLKEMDDMDPEKFADALCYAAKVERARARKLAAVRRLCSVTAALKAKAAELMAALEEDPLAAQETAAQLKAERDYLNTELERANAAKAEELEAAKKALTQAFNTRFQKAKTRWLAEKENAYILGMKECRSQFFLTARGHQFLRIMLNDTLEAFCRTPESLDVLGPGMGHLINDVCTLLMDQIGATPEQRALYDFDQIVDSVDAEGLDRVLGIDSQAPRTPAWWAPVVDKALKQFVEGTCADPLPTSPLLRTPYLDRLQAAAATRLNAEAAAGEPLFYPRPPAAAMTGAAAMELQASATHDHDQLRDAMLC
SmHDA2	SMILT020074.1	MSRSSNDSVSTRNQRVGLIFDERMYKHHAPHEKDHVECPDRIRVIWDLLNSSGLAKRCIILKAKAAEDNHLALVHTKNHITLIKNISSKNTKSKRKRIAEKFDSIYFNEGSTEAACLAAGSVIEAAEKVAKGEIDSAFAIVRPPGHHAEEGEPMGFCLYNNVAIAASYLLNERQELGINKILIVDWDVHHGNGTQKMFYKDPRVLVFSVHRHDFGTFYPTGDDGSYIMTGEGAGAGHNINVPWEHGKCGDADYLAVWDHILIPITKEFEPDMIIISAGFDAAKGDPLGGCCVSPNGYSIMLHKLMKFAGGKIVMSLEGGYNLNSTACSVRACVEVLLHEKSPNVVLAANPFDSTWSVILDVGRVLRPYWACDLLDLDKLWTFMGSSDDQDLIDFNEFSTVLGSEQNLPALAQEKKTTTLEQLGSLMPEPSIAKKTSTLSLAEPSDTEPLSKRLKSKASTTKRVLTYSPEISSPTNQPTSSPFKQISAPHSSDNPLSVVPIQQTLPETVVQSPTPNDQIMPTLPPRQPSPPQQTPAHISALISPEPPISQEKQSDNIEQPVLHSDQATSPHVSKKSSGKKKCRDAQGPSSEPGAKKSWPSVSPVPQKGSQTKIQLPGSLLKLNPAGSWAFVRSLTSSHDLAKLTSAECWAVLDALTSSKDVAMLRELSTEQLVDMICFNLVEQIILVSEVGSRLESCEEKDAALKELKSEVKEKDVKLVKTRNLNAKLEAKNRELQQRLANAEKENEALTMKARAEGKEDGIKLCRERILMALDRPF
SmHDA3	SMILT020076.1	MESNGEIESPLSRSNDGVSGKDRRVGLIFDERMCKHYAPAEEDHPESPDRIRVIWDLLNSSGLAKRCILLKAKDAEDNHLAL

		VHTKNHISLIKSISSGNSGSKRKRIAAKFNSIYFNEGSTEAA<br>CLAAGSVIEAAENVAIGEIDSAFAIVRPPGHHAEEGEPMGF<br>CLYNNVAIATSYLLNERRDLGIKKILIVDWDVHHGNGTQK<br>MFYKDPRVLFFSVHRYDFGTFYPSGHDGSYIMTGEGPGAG<br>HNINVPWEHGRCGDADYLAVWDHILIPVAKEFNPDMIIISA<br>GFDAAIGDPLGGCRISPYGYSIMLDKLMEFAGGKIVMALE<br>GGYNLNSIASSARACVEVLLHDKPPNVLSEAYPFASTWRVI<br>QEVRRELSTYWPVLAGDLPEKMISKTSQIEILSSDSEAETEE<br>ITLAVPEEVQTIEDIIQPLSNLKIDLDSRDQEATVKPSWRSEL<br>SKVDIWYATYGSNMKMSRFCCYIEGGQIEGMMRPCVGSM<br>DKSKPKEIIWKTFPHRLFFARERTATWGPGGVAFLHPTSNV<br>QEKTYMCLYKITLEQFNDVLLQENISNQEMSRPLFDSTALQ<br>TIQTEKCISVELVQRGWYHNVVYLGKEGDIPILTMTCSLSD<br>VDNFLAGVFPINPPCKEYANTLIKGLVEGKQLSEEEASAYIQ<br>EASSKHL
SmHDA4	SMILT031105.1	MNHFSPSEIELLNGKVIYSVAAAMGHNKESHPECNSRVPAII<br>DALEKMKLTSEFRGMDIIELEKFRPAKVEDIARVHERAYVS<br>GLEKAMDQASEQGLILIEGSGPTYATSTTFQESLLAAGAGIS<br>LVDAVVAASKIRKDPPVGFALVRPPGHHAVPTGPMGFCVF<br>GNVAIAARYAQRAHGLKRIFIIDFDVHHGNGTNDAFYDDP<br>DIFFLSTHQDGSYPGTGRINQIGRGDGEGATLNLPLPGGSG<br>DRAIRDVFEEVIAPCAQRFKPDIILVSAGYDAHVLDPLANL<br>QFTTSTYYMLASSIQQLARDLCGGRCVFFLEGGYNLTSLSH<br>SIADSFRAFLGEPSLAAEFDNPAFLYEEPSKRVKEAIQRVKH<br>IHSL
SmHDA5	SMILT012722<br>.1	MDTGGNSLPSGADGSKRKVCYFYDPEVGNYYYGQGHPM<br>KPHRMRMTHALLAHYGLLQNMHVLKPNPARDKDLCRFH<br>ADDYVSFLRRITPETQQDQLRQLKRFNVGEDCPVFDGLYS<br>FCQTYAGGSIGGAVKLNHGHCDIAINWAGGLHHAKKCEAS<br>GFCYVNDIVLAILELLKTHERVLYVDIDIHHGDGVEEAFYIT<br>DRVMTVSFHKFGDYFPGTGDIRDIGFGKGKYYALNVPLDD<br>GIDDESYQSLFKPIISKVMEVYRPGAVVLQCGADSLSGDRL<br>GCFNLSVKGHAECVRFMRSFDVPLLLLGGGGYTIRNVARC<br>WCYETGVALGIEIDDKMPQHEYIEYFGPDYTLHVAPSNME<br>NKNCRDMLEEIRAELLKNLSRLQHAPSVQFQERPPDIELPE<br>VDEDEENKDERKRPDSNVNIDYERNSTPSRVKMEFVEPMA<br>NDKNDEAVDELARKIDSTLAPTSSKGSMAAAEVDGMQYV<br>KLEKENTKTLLDELAPMDQH
SmHDA6	SMILT011069.<br>1	MDTGGNSLASGADGVKRKVSYFYDPEVGNYYYGQGHPM<br>KPHRIRMTHFLLAHYGLLQHMHVLKPNPARDKDLCRFHA<br>DDYVSFLRSITPETQQEQLRQLKRFNVGEDCPVFDGLYSFC<br>QTYAGGSVGGAVKLNHGHCDIAVNWAGGLHHAKKCEAS<br>GFCYVNDIVLAILELLKVHERVLYVDIDIHHGDGVEEAFYT<br>TDRVMTVSFHKFGDYFPGTGDVRDIGYGKGKYYSLNVPL

		DDGIDDESYQSLFKPIMGKVMEIFKPGAVVLQCGADSLSGDRLGCFNLSIKGHAECVKFMRSFNVPLLLLGGGGYTIRNVARCWCYETGVALGIELDDKMPQHEYYEYFGPDYTLHVAPSNMENKNSRHLLEEIRSKLLDNLSRLQHAPSVQFQERPPDSELPQMEEDHDGEDERYDPDSDMDIDDERKPLPGRVKSEFPEPEPKDMDDAKEDEPNREVDLKCSEPLA
SmHDA7	SMILT025982.1	MKGTAGDVGNVYFGPNHPMKPHRLCMTHHLVLAYELHNKMEIYRPHKAYPVELAQFHSPDYVEFLQRITPDKQVLFANEMAKYNLGEDCPVFENLFEFCQIYAGGTLDAARRLNNRLCDIAINWAGGLHHAKKCEASGFCYINDLVLGILELLKYHARVLYIDIDVHHGDGVEEAFYFTDRVMTVSFHKYGDLFFPGTGDVKDVGEKEGKNYAINVPLRDGIDDGSFLRLFKTIILKVVECYAPGAIVLQCGADSLAGDRLGCFNLSIDGHAACVKFVKQLNLPLLVTGGGGYTKENVARCWTVETGALLGVELASEIPENEYFKYFAPDYSLKCASGHMENLNSKSYLNNIRQQVCENLSAIQHAPGVQMQEVPPDFYIPDFDEDEQNPDERVNRHLQDKQVQRDDEYYEGDNDNDQNNDDS
SmHDA8	SMILT016201.1	MSRFKEEEIGTRLRNGDSAPLAEAASSNSFKPHLAEQLMKQKNGKRKSDMSLEEMYNSQYDFGDDEDDDSDWEPSALPAHSVVEIPKWFCLNCTMLNIGDDSHCDVCGEHRESGILKRGFVSSASSREVVTQNGAHAADGLQDPHPLMQSIASDNPTAIGFDERMLLHEEVVMKSHPHPERPDRLRAIAASLATTGIFPGRCHPISAREITQEELLKVHSLENIEAVEITSRVFSSYFTPDTYANQYSATAARLAAGLCFDLAAAIWSGRAKNGFALVRPPGHHAGVKHAMGFCLHNNAALAASAAQIAGAKKVLIIDWDVHHGNGTQEIFEKNKSVLYISLHRHEGGKFYPGTGSAYETRNIILVPVTFICEGKWYSFQLVDVANLFHMTGSMGAEGHCVNIPWSRGGVGDNDYIFAFQHVVLPIATEFDPDFTIISAGFDAARGDPLGGCDVTPAGYAQMTQMCRALSGGKVLVILEGGYNLRSISSSATAVIEVLLGESPKQNVGQVMPSKAGVRAVLEVLKIHLNYWSSLESKFTKLQSEWGWYTLEDKS
SmHDA9	SMILT029344.1	MDSDGGASLPSSCPDARKRRVSYFYEPTIGDYYYGQGHPMKPHRIRMAHNLIVHYSLHRRMEISRPFPAAFGDIRRFHSPEYVEFLSSVSPDTLHDHTHARHLRRFNVGEDCPVFDGLFNFCQASAGGSIGAAVKLNRQDADIAINWAGGLHHAKKSEASGFCYVNDIVLGILELLKVHRRVLYVDIDIHHGDGVEEAFYVTDRVMTVSFHKFGDFFPGTGHIKDIGVASGKYYALNVPLNDGLSDDNFRSLFRPILEKVMGVYQPDAVVLQCGADSLSGDRLGCFNLSVKGHADCLRFLRSFNVPLMTAVAVDVEPDNKLPYNEYYEYFGPDYTLHVEPSNMENLNTLRDLEKIRNMLLDQLSKLQHAPSTQFQTMPPTTEVPEEREEDMEVRPKPRIWNGDLGYYESDEDEIDKPTVRRSLNNEDAYGENTDLRSRTLSNNQLKLPHPPRK

SmHDA10	SMILT005242.1	MADEVIHVFWEEGMLRHDAGRGVFDSGLDPGFLDVLEAHPENAGRVKNMVSILKRGPIAPFLSWHQGRRALISELLSFHTQEYIDELVEADRSGGKDFGGGTFLNPGSWDSALLAAGTTLSAMKHILDGNGKVSYALVRPPGHHAQPTRADGYCFLNNAGLAVELALSSGVSKVAVIDIDVHYGNGTAEGFYRSNRVLTISLHMNHGSWGPSHPQNGTVDELGEGEGYGYNLNIPLPNGSGNRGYAHAVTELVVPAIERFDPEMMVLVVGQDSSAFDANGRQCLTMEGYRKIGQIVGEMAEKHSAGRVLIVQEGGYHLTYSAYCLHATLEGVLGFPAPLLSDPLGCYPEDEAFTVAVVQSINKFHRESVVPFLK
SmHDA11	SMILT020075.1	MKKMSRSSKDCVSGRDRRVGLIFDERMYKHYAPHEKDHVECPDRIRVIWDLLNSSGLAKRCIILNAKAAEDNHLALVHTKNHITLIKNIKKVAKGEIDSAFAIVRPPGHHAEEAEPMGFCLYNNVAIAASYLLNDRQELGINKILIVDWDVHHGNGTQNMFYKDPRVLVVSVHRHDFGTFYPTGGDGSYFMTGEGAGAGHNINVPWEHGQCGDADYLAVWDHILIPVAEEFKPDMIIISAGFDAEALQSRVNEMDAELKKTRNLNAKLEAEKLELQQRLGNAEKGKEIVRMKARAEGKEEGIKLCRERILMPF
SmHDA12	SMILT030323.1	MSSVPTRRHVAPIHGTSPPIKSSATLLHLYNFRHSIKIAFSGDMAATSSSRGESSDTAASVSHGGESVRRNRILASKLYFDVPASKIPLIYSSSYDIAFLGMEKLHPFDSSKWGRICRFLIAEGLLDKKHIVEPLEAKRDDLLVVHPESYLDSLKSSLNVATIVEVPPVALLPNCLVDKNVLHPFRKQVGGTILAAKLAKERGWAINVGGGFHHCSAQKGGGFCAYADISLCIQFAFTRLDISRVMIIDLDAHQGNGHEKDFSDDRRVYILDMYNPAIYPLDYEARRYITQKVEVACGTNTDEYLSKLDGALKVAGGAFDPELIIYNAGTDILDGDPLGKLKISPEGIATRDEKVFTFARGYMKSSARVIADSIINLSKKSLITIGDDQNDTIT
SmSRT1	SMILT012742.1	MYETSLNELELTEPKRARVRLRFNNRAGPSRARAHRICNRVELELKNLKLDRVELELELDTYRPSSSSKTVRSISMNKETFWRPRMISFQGSLKFVHTYRITPTGATMENKELPSNYLKDKMMVPNADPPRDEDVNLLSQFFKRSSRLVILTGAGISTESGIPDYRSPNGAYSTGFRPITHQEFMRSSRARRRYWARSYAGWRKFTTAQPGPAHIALASLEKAGRVKFMMTQNVDRLHHRAGSNPLELHGTVYIVACTNCGFSLSRSSFQDQVKAINPKWAEAIESLDYDSRSDKSFGMKQRPDGDIEIDEKFWEEEFHIPNCEKCDGILKPDVVFFGDNVPKGRSDRAVEAAKECDAFLVLGSSLMTMSAFRLIRAAHEAGAATAIVNIGVTRADDFVPLKINSRLGEILPRLLSVGSLGVPVV
SmSRT2	SMILT009140.1	MSLGYAEKLSFIEDVGNVGMAEFFDPPHVFQEKVERLAGMIQKSKHLVVFTGAGISTSCGIPDFRGPKGIWTLQRQGKALPEASLPFHRATPSTTHMALVEFEKAGILKFLISQNVDGLHLRSGIPREKLSELHGNSFMEQCPSCGAEYVRDFEIETIGLKETTRRCSKAGCGAKLRDTVLDWEDALPPKEMDPAEKHCKM

		ADLVLCLGTRFCSWPKISPGKITPACNLPLKCLKGGGKIVIV NLQKTPKDKKASLVIHGRVDKVMTGVMDMLSMRIPPFIRI DLFQIIITQALSLDKKYVNWTLRIASVHGKQAPLPFIKSVEV SFSESLSMKAAVLYDQPLHLKRRTAKSTKPFDVFLNLNFSA GCKCTYADLKIPVDFEIPTDCLKEDKDSIIEKLKERAVREPC CGQTAVVERRGILVPKSEVIVHAIVTNIIEYGGSLGASSLSN GSLKRRNEGLIDSGVCWKRTKARKRTSRHRR
SmHDT1	SMILT015039.1	MEFWGVEVKVGEKLKVQPKPGKLIHISQAAMGEVKDVK AAKNVHLRMKIDDKDFIIGSLAAEIRPQLMFDLVFEKDFEL SHDLKNGSVHFMGYIADDPEDEVEFSDFGSESADEEEPIEA KEEAKENGKAKARSVDAKPAAAAGKAHKEEKVAPKVEE DSDDDEDDSDDEMAFDTSDLSGDSDDEEDEDSSSSEEELP VAKQQQQSKKRPAASAEKSPAAKKAKSATPDKSGKKGQN ATPFPAKSAGKTPNKPKEQSPKTGGHATGKPFNKNFSGKG KGKHGGK
SmHDT2	SMILT013286.1	MAFWGIEVKPGKPYILSDEEKGRLHVTQATLGSGSSTKKSI LQCEVGDKKPIYLCSLLPDRLETCPLNLEFEEDEKVTFSIIG PQSVHLSGFFYGDESDDEDDEDGYGCGLYEDDAMGIGSE DEEDADYDSEDEEDDEDLSEDDLCCGYPHSPVPNSGVKIE EIVDDGKPTNENGMSKRAKKKKTDGNDNSNSQIVPKAGT SVPVLESEDEDGFPVPAHDKKSEANLEKTKQESSGKKSQN KSEKTDAASGRNLKRKSGAVNQDEQPASDIEPHSSSAQPD TTTNEVKQKKNKKKKVVQKLDSSLENEKPDSDGMKESPV AEAGNDLKPSSEKKKDKKKKKQNKLQENTPTPSAEKKVA DKNESNLEKQEKGDASKSLQVRTFPNGLVIEELVMGRPDG KRASPGKKVGVHYIGKLKKNGKIFDSNVGTAPFKFRLGIG QVIKGWDVGVNGMRVGDKRRLTIPPAMGYGAKGCPPAIPP NSWLVFDVELVDAN

Table S2 Protein sequences used to construct the phylogenetic tree

Gene name	Gene ID
Salvia miltiorrhiza	
SmHDA1	SMILT033186.1
SmHDA2	SMILT020074.1
SmHDA3	SMILT020076.1
SmHDA4	SMILT031105.1
SmHDA5	SMILT012722.1
SmHDA6	SMILT011069.1
SmHDA7	SMILT025982.1
SmHDA8	SMILT016201.1
SmHDA9	SMILT029344.1
SmHDA10	SMILT005242.1
SmHDA11	SMILT020075.1
SmHDA12	SMILT030323.1
SmSRT1	SMILT012742.1
SmSRT2	SMILT009140.1
SmHDT1	SMILT015039.1
SmHDT2	SMILT013286.1
Arabidopsis thaliana	
AtHDA2	At5G26040
AtHDA5	At5G61060
AtHDA6	At5G63110
AtHDA7	At5G35600
AtHDA8	At1G08460
AtHDA9	At3G44680
AtHDA10	At3G44660
AtHDA14	At4G33470
AtHDA15	At3G18520
AtHDA17	At3G44490
AtHDA18	At5G61070
AtHDA19	At4G38130
AtHDT1	At3G44750
AtHDT2	At5G22650
AtHDT3	At5G03740
AtHDT4	At2G27840
AtSRT1	At5G55760
AtSRT2	At5G09230
Oryza sativa	
OsHDA713	LOC_Os07g41090.3
OsHDA704	LOC_Os07g06980.2
OsHDA709	LOC_Os11g09370.1
OsHDA714	LOC_Os12g08220.1
OsHDA702	LOC_Os06g38470.3

OsHDA705	LOC_Os08g25570.1
OsHDA710	LOC_Os02g12380.3
OsHDA703	LOC_Os02g12350.1
OsHDA701	LOC_Os01g40400.1
OsHDA712	LOC_Os05g36920.1
OsHDA716	LOC_Os05g36930.2
OsHDA711	LOC_Os04g33480.1
OsHDA706	LOC_Os06g37420.1
OsHDA707	LOC_Os01g12310.1
OsSRT702	LOC_Os12g07950.2
OsSRT701	LOC_Os04g20270.1
OsHDT701	LOC_Os05g51830.1
OsHDT702	LOC_Os01g68104.1
Solanum lycopersicum	
SlHDA1	Solyc09g091440
SlHDA3	Solyc06g071680
SlHDA2	Solyc03g112410
SlHDA4	Solyc11g067020
SlHDA5	Solyc08g065350
SlHDA9	Solyc03g115150
SlHDA8	Solyc03g119730
SlHDA7	Solyc01g009110
SlHDA6	Solyc06g074080
SlHDA10	Solyc01g009120
SlSRT1	Solyc07g065550
SlSRT2	Solyc04g009430
SlHDT2	Solyc10g085560
SlHDT3	Solyc11g066840
SlHDT1	Solyc09g009030
Zea mays	
ZmHDA101	Zm00001d053595
ZmHDA102	Zm00001d003813
ZmHDA103	Zm00001d018346
ZmHDA104	Zm00001d016381
ZmHDA105	Zm00001d014076
ZmHDA106	Zm00001d050139
ZmHDA107	Zm00001d024221
ZmHDA108	Zm00001d039202
ZmHDA116	Zm00001d046388
ZmHDT101	Zm00001d042433
ZmHDT102	Zm00001d011139
ZmHDT103	Zm00001d032210
ZmSRT101	Zm00001d019276
ZmSRT102	Zm00001d023585

ZmSRT103	Zm00001d025705
ZmSRT104	Zm00001d046205
ZmSRT105	Zm00001d047121

Table S3 Gene ID used to construct a co-expression network.

Gene name	Gene ID in PRJNA393563	Gene ID in PRJNA703309
SmPAL1	SMil_00019885	TRINITY_DN23917_c0_g2
SmTAT1	SMil_00024924	TRINITY_DN15459_c0_g1
SmC4H1	SMil_00000716	TRINITY_DN18900_c0_g1
SmHPPR1	SMil_00002680	TRINITY_DN14918_c0_g1
Sm4CL1	SMil_00008129	TRINITY_DN20844_c0_g1
SmRAS1	SMil_00025190	TRINITY_DN23540_c0_g1
SmHDA6	SMil_00014906	TRINITY_DN23308_c1_g1
SmMYC2	SMil_00026019	?
SmJAZ9	SMil_00002123	?
SnRK2	?	TRINITY_DN7280_c0_g1
PP2C	?	TRINITY_DN23712_c2_g1

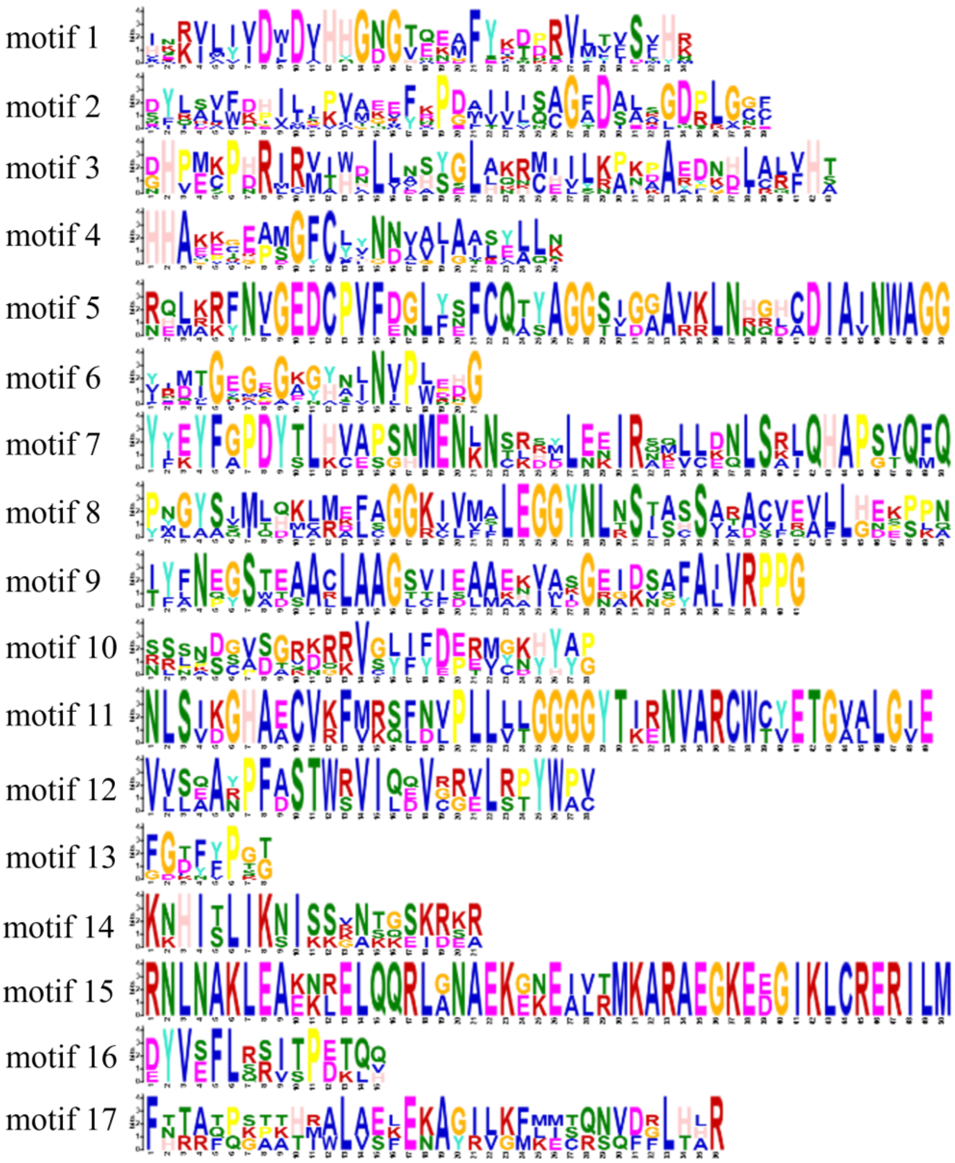

Figure S1 Seventeen motif logos in SmHDAC protein
